# Supplementary figures and images for: Effect of thermal cycling on the mechanical properties of conventional, milled, and 3D-printed base resin materials: a comparative in vitro study
Source: PeerJ. 2025 Mar 17;13:e19141. doi: 10.7717/peerj.19141 (PMC11925046; doi:10.7717/peerj.19141)

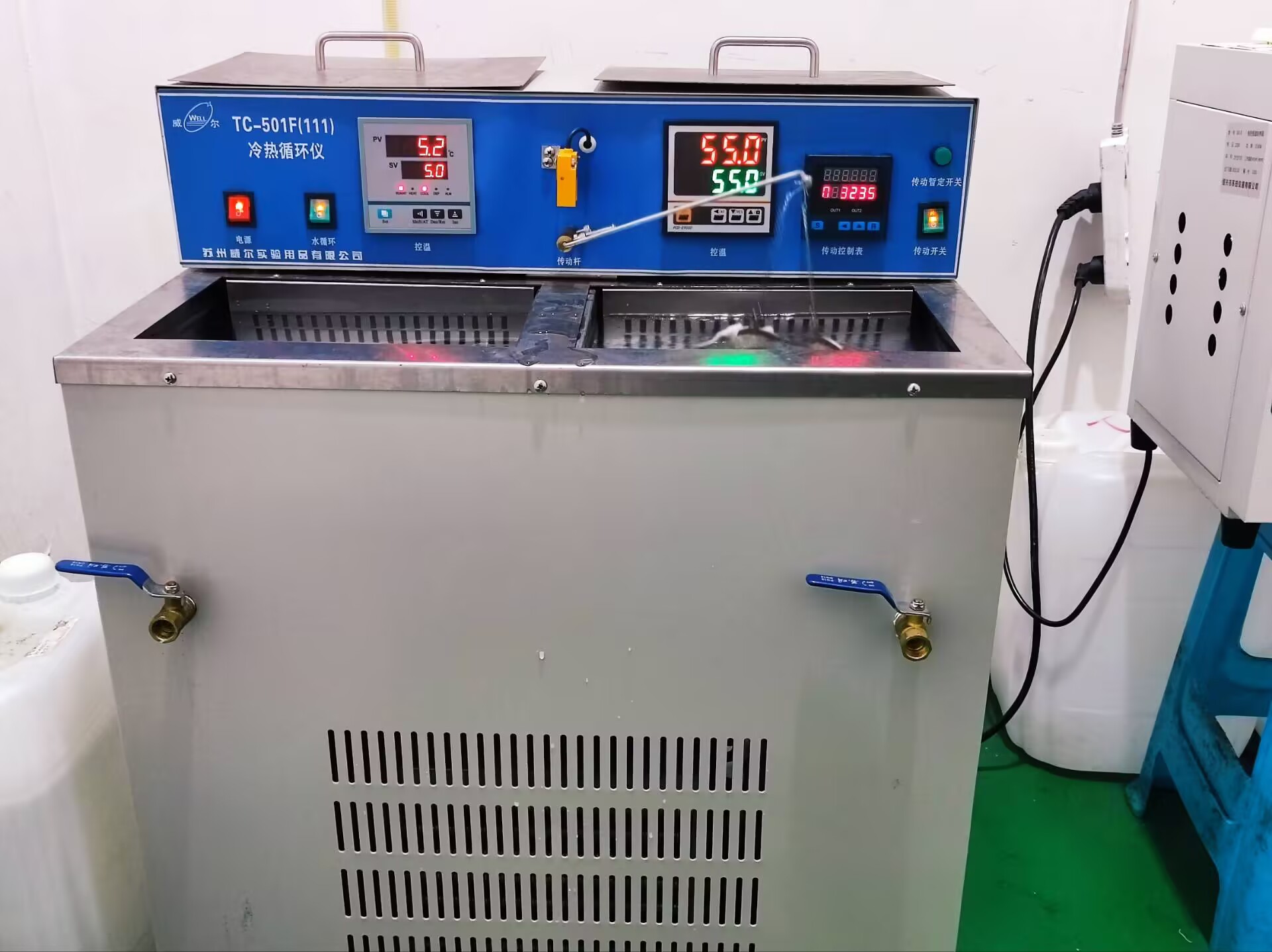

Supplement: Supplemental Information 2 [file peerj-13-19141-s002.jpg]

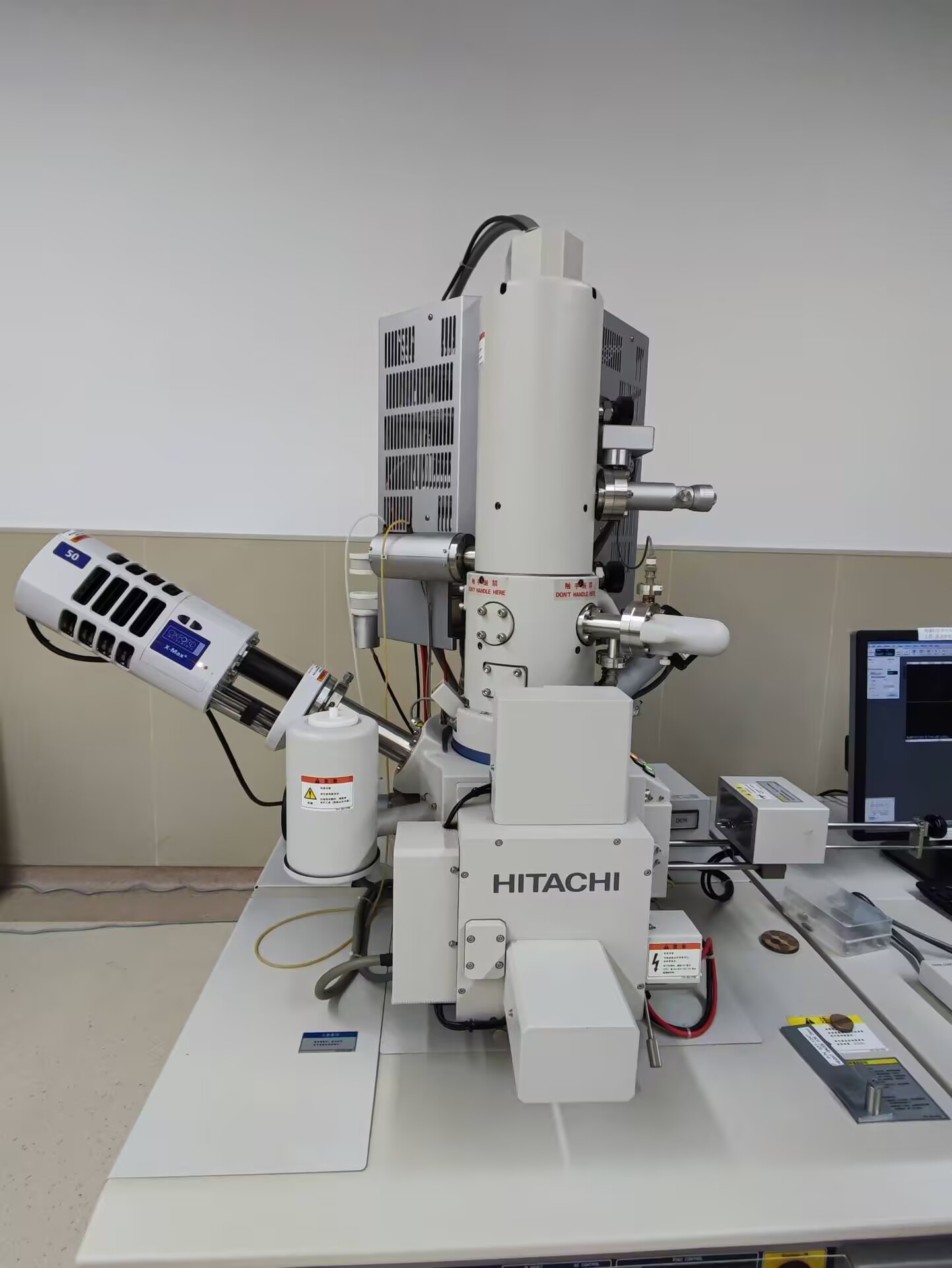

Supplement: Supplemental Information 3 [file peerj-13-19141-s003.jpg]
